# Clinical characteristics and management strategies in adult foreign-body airway obstruction: A retrospective cohort study

**DOI:** 10.1371/journal.pone.0351214

**Published:** 2026-06-05

**Authors:** Hongzhen Yin, Tong Wang, Changshun Zhong, Yingya Cao, Xiaogan Jiang, Qiancheng Xu, Weihua Lu

**Affiliations:** 1 Anhui Medical University, Hefei, Anhui, China; 2 Department of Critical Care Medicine, The First Affiliated Hospital of Wannan Medical University (Yijishan Hospital of Wannan Medical University), Wuhu, Anhui, China; 3 Anhui Provincial Clinical Research Center for Critical Respiratory Disease, ‌‌Wuhu, Anhui, China; 4 Perioperative Monitoring and Prognostic Technology Research and Development Center of Wuhu, Wuhu, Anhui, ‌‌China; The University of Kansas Health Systems St. Francis Campus, UNITED STATES OF AMERICA

## Abstract

Airway foreign-body aspiration in adults is uncommon but can be life-threatening.Flexible bronchoscopy is the standard first-line therapy,but critically ill patients may need extracorporeal life support.This study aims to characterize the diagnosis,management,and outcomes of adult airway foreign-body cases treated at a single center over nearly 12 years to inform a standardized clinical pathway.A single-center retrospective observational study of consecutive patients aged ≥14 years with confirmed airway foreign body who were treated at a tertiary hospital in China were conducted.Medical records of consecutive adolescent and adult patients diagnosed with airway foreign-body aspiration and admitted to the hospital from 01/01/ 2014–30/11/2025 were reviewed.Data included demographics,imaging,extraction method, respiratory support and so on.Descriptive statistics were reported as medians with interquartile ranges or counts and percentages.A total of 41 patients were included,with a median age of 59.5 years(interquartile ranges 51–72) and 65.85% male.Flexible bronchoscopy was attempted as the primary intervention in 38 patients(92.68%) and succeeded in 81.58%(31/38) to remove airway foreign body.Most patients(78.05%) required only nasal cannula oxygen,while nine patients(21.95%) needed advanced support including mechanical ventilation (14.63%),high-flow oxygen(4.88%),and extracorporeal life support (2.44%).At discharge,most survivors had a good neurological outcome,with 36 patients(87.80%) having a Cerebral Performance Categories score of 1.The 28-day survival rate was 92.68%.These findings show that flexible bronchoscopy is an effective first-line therapy,and rigid bronchoscopy or surgery is useful when flexible bronchoscopy fails.In unstable cases,timely extracorporeal life support can bridge to definitive removal.These results support a tiered,multidisciplinary approach incorporating early chest computed tomography,flexible bronchoscopy,and escalation to advanced airway or extracorporeal support.

## Introduction

Airway foreign-body aspiration in adults,though less common than in children, constitutes a significant clinical emergency that can lead to substantial morbidity and mortality [1].The incidence is highest among older adults, particularly those with underlying neurological deficits,impaired swallowing or cough reflexes,or psychiatric conditions [1,2].The immediate risk of asphyxiation and long-term complications such as recurrent pneumonia,bronchiectasis,and lung abscesses underscore the need for rapid diagnosis and effective intervention.Diagnosis primarily relies on a suggestive patient history and radiological findings,with the history of an aspiration event being the most crucial clue for prompt medical consultation [3].Traditionally,the mainstay of treatment for airway foreign body（AFB） has been bronchoscopic removal.Rigid bronchoscopy has long been considered the gold standard,demonstrating high success rates,particularly for large or impacted objects [4].However,recent trends indicate a significant shift towards the use of flexible bronchoscopy,which is now often employed as a primary intervention tool due to its minimally invasive nature,wider accessibility, and diagnostic capabilities [3,5].The choice between rigid and flexible bronchoscopy is often dictated by the characteristics of the foreign body,its location,and the clinical stability of the patient,creating a complex decision-making landscape for clinicians [5].

Furthermore,recent advancements in interventional techniques and advanced life support have begun to reshape the management of high-risk AFB.Procedural complications during bronchoscopic retrieval,such as hypoxia,airway trauma,or failure to extract the object, contribute significantly to mortality [6].In this context,veno-venous extracorporeal membrane oxygenation(VV-ECMO) has emerged as a life-saving bridge,providing essential gas exchange and hemodynamic support for patients with critical airway obstruction [7–9].The use of ECMO allows for a more controlled and unhurried environment for complex bronchoscopic interventions,mitigating the risk of intraprocedural hypoxemia and respiratory collapse [10,11].

Despite these technological and procedural advances,there remains a lack of standardized, integrated workflows for managing adult AFB,especially in severe cases.The decision of when to transition from flexible to rigid bronchoscopy,or more critically, when to proactively initiate ECMO support,is often based on institutional experience rather than evidence-based guidelines. To improve patient outcomes,it is imperative to optimize the management process,from initial assessment to the judicious application of advanced support technologies.This retrospective study reviewed adult AFB cases over nearly 12 years at our institution,analyzing the diagnostic and therapeutic strategies employed across different departments.By summarizing these experiences,we aim to identify current trends,highlight challenges,and propose an optimized management algorithm to provide a clearer,more effective framework for clinicians.

## Methods

This single-center,retrospective observational study was conducted at Yijishan Hospital of Wannan Medical College,a tertiary academic medical center in Wuhu, China.Medical records of consecutive adolescent and adult patients diagnosed with AFB who were admitted to the hospital from 01/01/2014–30/11/2025 were reviewed.The study was approved by the Institutional Review Board of the Scientific Research and New Technology Department of Yijishan Hospital of Wannan Medical College (Approval No. 2025-ky-306) on 09/12/2025 and was conducted in accordance with the Declaration of Helsinki (as revised in 2013).Given the retrospective design,individual informed consent was waived.Data for this study were accessed for research purposes from 10/12/2025–20/12/2025.

### Inclusion criteria

- Age ≥ 14 years;
- Foreign body located in the supraglottic region,glottis,subglottic area,trachea,or bronchial tree,confirmed by at least one of the following:

1. Radiological evidence of the foreign body on neck or chest imaging (X-ray, chest computed tomography),
2. Endoscopic visualization during laryngoscopy or bronchoscopy,
3. Observation of expelled foreign body during coughing.

### Exclusion criterion

- Foreign body located outside the airway(e.g., esophagus);
- Planned placement of airway devices or materials (e.g., tracheostomy tube, endobronchial stent)not related to aspiration;
- Incomplete hospitalization records preventing extraction of key clinical variables.

### Data collection

A comprehensive review of the hospital’s electronic medical record system,the bronchoscopy database,and the Picture Archiving and Communication System was conducted to identify eligible cases and extract data.Two investigators independently collected the data to ensure accuracy,and any discrepancies were resolved by a third senior investigator.

The following variables were collected for each patient:

- **Demographic and clinical data:**age,sex,admitting department(e.g.,Respiratory Medicine,Thoracic Surgery,Intensive Care Unit),pre-existing comorbidities (with a focus on neurological conditions such as cerebrovascular disease,Parkinson’s disease,traumatic brain injury,central nervous system infection and dementia),and chief presenting symptoms(e.g.,cough,dyspnea,choking,hemoptysis,asphyxia).
- **Radiological data:**type of imaging modality used(chest X-ray,chest computed tomography with or without three-dimensional reconstruction) and the rate of AFB detection by chest computed tomography(CT).
- **Procedural details:**the primary method of AFB extraction(flexible bronchoscopy,rigid bronchoscopy,or surgery),and the highest level of respiratory support required during hospitalization(nasal cannula,high-flow nasal cannula,non-invasive ventilation,invasive mechanical ventilation,or VV-ECMO).
- **Foreign body characteristics:**the nature of the AFB (categorized as bony,organic plant-based,food bolus,medical-related,or inorganic non-medical) and its precise anatomical location within the tracheobronchial tree.
- **Clinical outcomes:**the success rate of bronchoscopic removaltotal length of hospital stay,length of ICU stay,28-day mortality and other outcomes.

### Statistical analysis

All data were entered into a spreadsheet(Microsoft Excel 2010, Redmond, WA, USA) and analyzed using SPSS Statistics(Version 25.0, IBM Corp., Armonk, NY, USA).Descriptive statistics were used to summarize patient characteristics.Continuous variables were presented as mean±standard deviation or median and interquartile range,as appropriate.Categorical variables were presented as frequencies and percentages(n, %).

## Results

### Patient characteristics

**Fig 1:**
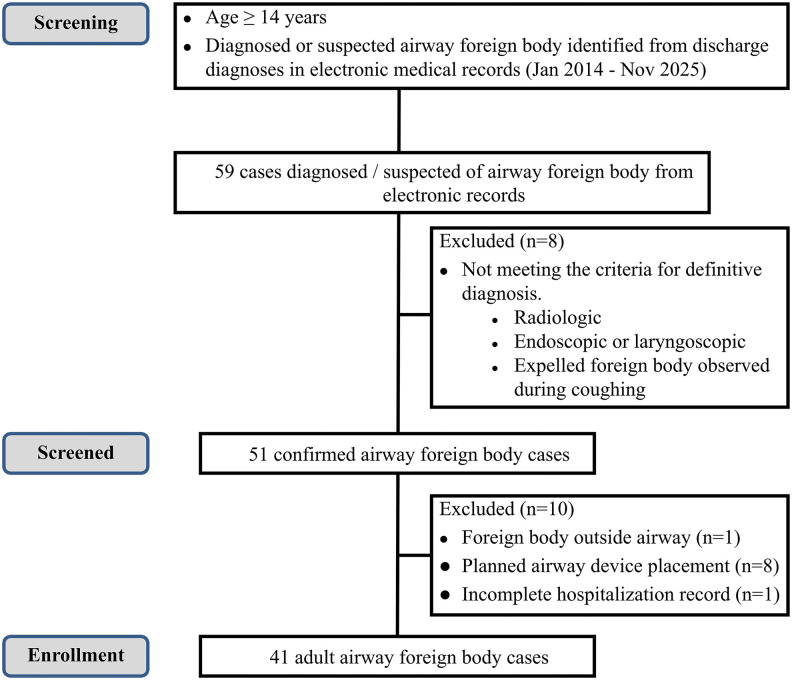
Flowchart of patient selection.

A total of 41 patients with foreign-body airway obstruction were included in this study (**Fig 1).These patients come from the Intensive Care Units (ICUs), including the Respiratory Intensiv**e Care Unit (RICU) and the Emergency Intensive Care Unit (EICU), as well as from the Department of Critical Care Medicine and from general wards such as Respiratory Medicine and Otolaryngology.Among the 41 patients, only one was 16 years old,the rest were 18 years or older.The demographic and clinical characteristics are summarized in Table 1.The median age of the cohort was 59.5 years(IQR, 51–72 years),and 27 patients(65.85%) were male.The majority of patients(n = 32, 78.05%) were over 50 years old.21.95%(n = 9) had underlying neurological conditions that could predispose them to aspiration.

**Table 1:** Baseline characteristics of adult patients with foreign-body airway obstruction.

Variables	Value
**Demographic characteristics**	
Sex (Male, %)	27 (65.85%)
Age (years, %)	59.5 (51-72)
14-17yr	1(2.44%)
≥18yr	40（97.56%）
**Department**	
ICU(RICU/EICU/Critical Care Medicine)	12 (29.27%)
Respiratory Medicine	25 (60.98%)
Otolaryngology Medicine	1 (2.44%)
Others	3 (7.32%)
**Possible causes**	
Alzheimer’s disease hypertension	1 (2.44%)
Cerebral infarction	3 (7.32%)
Cerebral hemorrhage	1 (2.44%)
Parkinson’s disease	2 (4.88%)
Brain trauma	1 (2.44%)
Central nervous system infection	1 (2.44%)
**Clinical symptoms and signs**	
Cough	26 (63.41%)
Chest tightness	9 (21.95%)
Hemoptysis	2 (4.88%)
Dyspnea	1 (2.44%)
Suffocation	5 (12.20%)
**Chest CT examination**	38 (92.68%)
CT examination positivity rate	38 (100%)

### Clinical presentation and diagnosis

The most common presenting symptom was cough(n = 26, 63.41%),followed by chest tightness(n = 9, 21.95%),suffocation(n = 5, 12.20%),and dyspnea (n = 1, 2.44%).Hemoptysis occurred in two patients(4.88%).Chest CT was performed in 38 of the 41 patients(92.68%).In all 38 cases where a CT scan was performed, the foreign body was identified,yielding a diagnostic detection rate of 100% (**Table 1**).

### Foreign body characteristics

**Fig 2:**
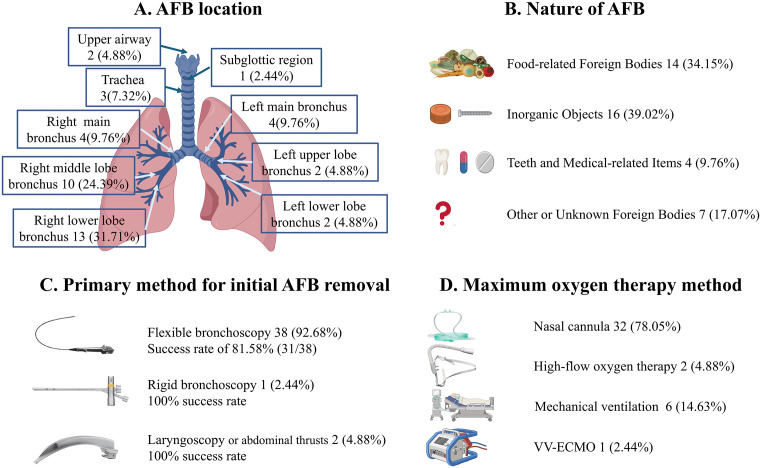
Anatomical location, composition, removal method, max respiratory support among 41 adults with AFB. **(A)** Anatomical distribution of AFB within the upper airway,tracheaand bronchial tree.The right bronchial tree was most frequently involved, particularly the right lower lobe bronchus (31.71%). **(B)** Nature of the AFB,categorized as food-related,inorganic,teeth/medical-related,or other/unknown.Inorganic objects accounted for the largest proportion (39.02%). **(C)** Primary method of AFB removal.Flexible bronchoscopy was performed in 92.68% of cases, with a procedural success rate of 81.58%(31/38) to remove AFB.Rigid bronchoscopy and laryngoscopy or abdominal thrusts achieved 100% success rate of AFB removal in their respective cases. **(D)** Maximum oxygen therapy required during hospitalization.Most patients were maintained on nasal cannula oxygen(78.05%),while 21.95% required advanced support;veno-venous extracorporeal membrane oxygenation(VV-ECMO) was used in one critical case.

The locations of the AFB are shown in Fig 2A.The right bronchial tree was the most common site of impaction(n = 27,65.85%),with the right lower lobe bronchus being the single most frequent location(n = 13,31.71%).The left bronchial tree was involved in 8 patients (19.51%).The nature of the retrieved AFB is shown in Fig 2B.Inorganic objects were the most common type(n = 16,39.02%),followed by food-related foreign bodies(n = 14,34.15%). Other types included teeth and medical-related items(n = 4,9.76%) and other or unknown foreign bodies(n = 7,17.07%).

### Treatment and clinical outcomes

Clinical outcomes are summarized in **Table 2**,and therapeutic interventions are detailed in Fig 2C-D.Flexible bronchoscopy was the most common removal method,performed in 38 patients(92.68%).Of these procedures,the AFB could not be removed in 7 cases,yielding an overall success rate of 81.58% for flexible bronchoscopy to remove AFB.The distribution of successful attempts among the 31 procedures was as follows:one successful attempt in 23 cases (60.53%),two successful attempts in 7 cases (18.42%),and three successful attempts in 1 case(2.63%).Rigid bronchoscopy was performed successfully in 1 patient (2.44%),and laryngoscopy or abdominal thrusts sufficed for 2 patients with AFBs in the upper airway(4.88%) (**Fig 2C**).

**Table 2:** Clinical outcomes of adult patients with foreign-body airway obstruction.

Outcomes	
Hospitalization proportion	(41/41) 100%
ICU admission proportion	(12/41) 29.27%
**Fiberscope bronchoscopy: initial usage**	(38/41) 92.68%
Success rate of AFB removal	(31/38) 81.58%
Number of successful attempts	
1	(23/38) 60.53%
2	(7/38) 18.42%
3	(1/38) 2.63%
Follow-up of the failed cases	(7/38) 18.42%
Total success rate of AFB removal	(4/7) 57.14%
Success rate of AFB removal by rigid bronchoscopy	(2/7) 28.57%
Success rate of AFB removal by fiberscope bronchoscopy	(2/7) 28.57%
Discharge with airway foreign body presence	(2/7) 28.57%
Deceased after self-discharge	(1/7) 14.29%
Mortality rate	(1/38) 2.63%
**Rigid bronchoscopy**	1 (2.44%)
Success rate of AFB removal	(1/1) 100%
Discharged after clinical recovery	(1/1) 100%
Mortality rate	(0/1) 0%
**Laryngoscopy or abdominal thrusts**	2 (4.88%)
Success rate of AFB removal	(2/2) 100%
Discharged after clinical recovery	(0/2) 0%
Mortality rate	(2/2) 100%
**Death or discharge not recovered**	(3/41) 7.32%
**General ward length of stay (29 patients)**	6.0 (2.0 - 8.0)
**ICU length of stay (12 patients)**	5.0 (1.5 - 10.5)
**Cerebral performance categories Scoring Scale**	
1	(36/41) 87.80%
2	(2/41) 4.88%
3	(0/41) 0%
4	(1/41) 2.44%
5	(2/41) 4.88%

Clinical analysis of the seven patients in whom AFB removal by bronchoscopy failed is summarized in **Table 2**.Removal by bronchoscopy failed in 7 patients(5 males and 2 females), with a disease course ranging from 1 month to 3 years in 6 patients,and the remaining one having a short disease course.The principal reason was failure to recognize the AFB in the acute phase,after which the AFB became adherent to normal tissue,complicating removal.Specifically, in 1 case the surface of foreign body was smooth,and it migrated deeper after detachment,precluding extraction.In another case,detachment was achieved but the object was too large to retrieve.It is recommended to transfer to an external center for rigid bronchoscopic retrieval.We followed up these 7 patients.Among them, 3 ultimately retained the AFB(1 left against medical advice, 2 discharged with minimal symptoms).Four patients underwent further treatment at other institutions.Removal was successful with rigid bronchoscopy under flexible-bronchoscopic guidance in 2 patients with large or smooth AFB.In the remaining 2 patients,removal was completed under flexible-bronchoscopic guidance after further sessions due to adhesions(**Table 2**).

Regarding respiratory support,32 patients (78.05%) were managed with a nasal cannula. Nine patients (21.95%) required advanced support,including mechanical ventilation (n = 6, 14.63%),high-flow oxygen therapy (n = 2, 4.88%),and VV-ECMO in one case (2.44%) (**Fig 2D**).Among the cohort,a 62-year-old male with diabetes mellitus and a prior hemorrhagic stroke presented with unexplained hypoxic respiratory failure lasting sereral hours.Initial oxygen saturation was 60%.After endotracheal intubation,saturation remained around 30–40%,but no useful history was obtained from the family.With life-threatening hypoxemia despite ventilation, emergent bedside VV-ECMO was initiated, increasing SpO2 to 94–100% and PaO2 to 76.2 mmHg.Chest CT suggested a density in the left main bronchus with left-lung obstructive inflammation.Flexible bronchoscopy identified a foreign body (peanut fragment) in the left main bronchu. Following stabilization,the patient was extubated,weaned from VV-ECMO,and transferred back for rehabilitation.

All patients were hospitalized,with 12 (29.27%) requiring ICU admission.The median length of stay was 6.0 days(IQR, 2.0–8.0) for patients in the general ward and 5.0 days(IQR, 1.5–10.5) for patients in the ICU.The 28-day survival rate was 92.68%(n = 38).Thirty-one patients (75.61%) were discharged after clinical recovery,while 7 (17.07%) were transferred to another hospital.Three patients (7.32%) died during hospitalization (n = 2，4.88%) or were discharged in a moribund state without clinical recovery (n = 1，2.44%).At discharge,the majority of survivors had a good neurological outcome,with 36 patients (87.80%) having a Cerebral Performance Categories score of 1 (**Table 2**).

## Discussion

A total of 41 patients with AFB obstruction were included in this study.Flexible bronchoscopy was the effective first-line therapy in most cases(92.68%),achieving an overall success rate of 81.58% to remove AFB,with a single-attempt success rate of 60.53%.For patients with larger AFB or in whom flexible bronchoscopic extraction failed, rigid bronchoscopy or surgical escalation was required as a backup intervention.In hemodynamically or respiratory-unstable cases,timely initiation of extracorporeal life support (ECLS) could serve as a bridge to definitive AFB removal.A defining characteristic of adult AFB is its association with conditions that impair protective airway reflexes.The median age of patients in our cohort was 59.5 years,and 21.95% presented with significant neurological deficits.This demographic profile is consistent with substantial evidence identifying advanced age and neurological impairment as primary risk factors [6,12].For instance,studies by Hewlett et al. and Blanco Ramos et al. documented mean patient ages of 50–60 and 48 years,respectively [6,13].Specifically within the geriatric population,Lin et al. observed that AFB often presents with nonspecific clinical features,leading to misdiagnosis in 82.4% of their cases and diagnostic delays of up to three years [14].This observation highlights the diagnostic unreliability of patient history.The classic “penetration syndrome” is frequently absent, and patient recall of a choking event is reported in approximately 50% of adult cases,with rates decreasing to as low as 29–30% in patients over 65 years of age [14,15].Although a witnessed choking event is a strong predictor(Odds Ratio 5.0) [15],its absence should not diminish clinical suspicion,particularly in high-risk groups.Given the potential ambiguity of the clinical presentation, radiological imaging is indispensable for diagnosis and procedural planning.This aligns with literature indicating that chest X-rays are normal in 14% to 47.5% of confirmed AFB cases [13,15].Indirect signs such as non-resolving pneumonia (30.6%),atelectasis (18.4%), or unilateral hyperinflation are common but non-specific findings [16,17]. Consequently, CT has become the gold standard diagnostic modality [15].CT can precisely delineate the foreign body’s size, morphology, and anatomical location, most commonly the right bronchial tree (65–71.5%) [13,14].CT demonstrated a 100% detection rate for abnormalities in our series,which is consistent with reported sensitivities approaching 100% [15].

Our data reflected the procedural evolution in AFB management.Historically,rigid bronchoscopy was the established gold standard,valued for its large working channel and superior airway control [13,15].However, mirroring a global trend,our institutional practice has transitioned to a “flexible-first” strategy.In our cohort,flexible bronchoscopy was the primary modality and achieved a success rate of 81.58% to remove AFB.This approach is supported by a large systematic review by Sehgal et al.,which reported a pooled success rate of 89.6% in adults, and other reviews citing success rates of 79–90% [6,13,16].The preference for flexible bronchoscopy is driven by its minimally invasive nature,wider availability,reduced requirement for general anesthesia,and enhanced access to distal and angulated bronchi [14,18].Nevertheless,this strategy does not render rigid bronchoscopy obsolete.The technique remains an indispensable component of a tiered management approach,reserved for cases of flexible bronchoscopy failure,large or proximally impacted objects,or scenarios requiring definitive airway control.The case reported by Gómez-Ramos et al.,underscores the necessity for a readily available escalation plan [17].Indeed,literature indicates that rigid bronchoscopy or surgical intervention is required in 3.8% to 15.6% of cases [6,13].

A particularly challenging subset of cases involves chronically retained AFB,often resulting from the aforementioned diagnostic delays.Over weeks to years, the foreign body incites a persistent local inflammatory reaction,leading to the formation of obstructive granulation tissue that can encapsulate the object [13].Furthermore, the foreign body acts as a nidus for the development of resilient bacterial biofilms,which are structured microbial communities encased in a self-produced matrix and notoriously resistant to both host defenses and antimicrobial therapy [19,20].This complex biological response,characterized by granulation,epithelialization,and biofilm formation,effectively embeds the foreign body into the bronchial wall.This process significantly complicates bronchoscopic removal, transforming a simple retrieval into a high-risk procedure fraught with potential for severe hemorrhage, airway perforation, and failure [21].This is clinically reflected in our cohort, where several of the 7 flexible bronchoscopy failures were associated with a prolonged disease course (up to 3 years).Successful management in such scenarios often necessitates advanced interventional techniques,such as cryotherapy,laser ablation,or argon plasma coagulation to debulk granulation tissue,and frequently mandates the superior airway control and instrumentation capabilities of rigid bronchoscopy [13,22].

A pivotal advancement in AFB management is the strategy for patients presenting with severe airway obstruction and life-threatening hypoxemia.Procedural complications, particularly intra-procedural hypoxia,remain a primary source of morbidity [2].Our experience with a case requiring veno-venous extracorporeal membrane oxygenation(ECMO) highlights its role as a “bridge to intervention” [10,23,24].The application of extracorporeal life support, such as ECMO or cardiopulmonary bypass,offers a profound physiological advantage by uncoupling systemic oxygenation and ventilation from the compromised native airway [10,11,23].This capability is especially vital in cases of life-threatening AFB where cardiac arrest is imminent [11,24].By proactively initiating ECLS in patients with refractory respiratory failure,clinicians can create a stable physiological environment, thereby enhancing the safety and efficacy of AFB removal [10,11,25].This study is subject to several limitations.Its retrospective, single-center design introduces potential for selection and information biases.The modest sample size of 41 cases precludes sophisticated statistical analyses.Furthermore, over a 12-year period, differences in the experience levels of the specialist physicians involved in treatment may have affected AFB removal outcomes.Despite these limitations,our study contributes valuable real-world data from clinical practice over nearly 12 years,reflecting common challenges and contemporary solutions in the management of adult AFB.

**Fig 3:**
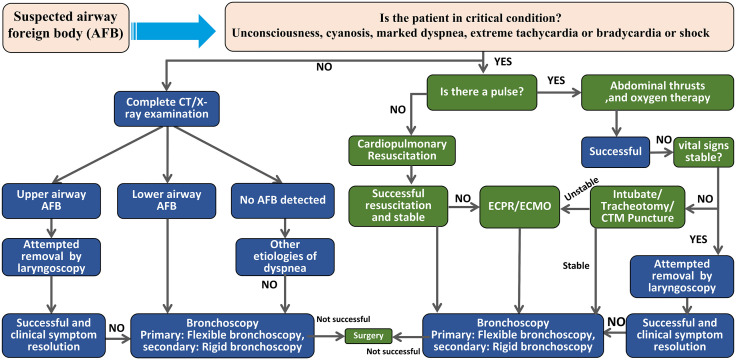
Management algorithm for adult foreign-body airway obstruction. Stable patients undergo imaging to localize the AFB, followed by removal via laryngoscopy or bronchoscopy with surgical backup. Unstable patients receive immediate airway rescue and oxygenation, progressing to CPR or extracorporeal life support (ECPR/ECMO) if indicated before definitive removal. Abbreviations: AFB: airway foreign body; CPR: cardiopulmonary resuscitation; ECPR: extracorporeal cardiopulmonary resuscitation; ECMO: extracorporeal membrane oxygenation; CTM: cricothyroid membrane.

Synthesizing these findings,the proposed management algorithm (Fig 3) provides a structured framework directly derived from our clinical data and the existing evidence.At the pre-laryngeal level,it emphasizes prompt basic life support measures and the Heimlich maneuver;if these are ineffective,immediate direct laryngoscopy is recommended as the first-line technique for AFB at or above the vocal cords,with particular attention to the risk of complete airway obstruction and sudden cardiopulmonary arrest.When laryngoscopy is negative or non-diagnostic and the patient’s respiratory and circulatory status has been stabilized or adequately supported,the algorithm transitions to intrathoracic evaluation.For clinically stable patients,it standardizes the use of chest CT,a step strongly supported by the 100% detection rate in our series and its value for procedural planning,and then advocates a “flexible-first” bronchoscopic strategy [13,16,17].This mirrors our institutional practice,in which flexible bronchoscopy was used in 92.68% of cases and achieved an 81.58% success rate of AFB removal.Within this bronchoscopic arm,the algorithm specifies that flexible bronchoscopy is preferred for most main airway foreign bodies, but that rigid bronchoscopy under flexible bronchoscopic guidance should be pursued when the object is too large,smooth, impacted,or otherwise difficult to retrieve safely with flexible bronchoscopy alone.Our 7 documented flexible-bronchoscopy failures,directly informed this predefined escalation pathway.For critically unstable patients at any level of obstruction,the algorithm prioritizes early aggressive resuscitation and incorporates ECLS not as a last resort but as a proactive tool; this is supported by our experience with a patient successfully bridged to intervention with VV-ECMO,in whom this strategy converted a time-critical emergency‌‌ into a controlled procedure and mitigated the risk of intraprocedural hypoxia [2,10,11].

## Conclusion

In conclusion, effective management of adult AFB necessitates a tailored, multidisciplinary strategy. While flexible bronchoscopy has emerged as a safe and effective first-line therapy, clinicians must be prepared to escalate to rigid bronchoscopy or surgical intervention when indicated.For the most critical presentations,integration of advanced life support techniques, particularly proactive use of ECMO, can support airway stabilization and facilitate safe foreign body removal.We believe the proposed algorithm offers a practical, evidence-based framework to guide timely decision-making and reduce the morbidity and mortality associated with this condition. Future prospective, multi-center studies are warranted to validate this ‌‌algorithm.

## Abbreviations

AFB: Airway foreign body
ECLS: Extracorporeal life support
VV‑ECMO: Veno‑venous extracorporeal membrane oxygenation
ICU: Intensive care unit
RICU: Respiratory intensive care unit
EICU: Emergency intensive care unit
IQR: Interquartile ranges
CT: Chest computed tomography
CPR: Cardiopulmonary resuscitation
ECPR: Extracorporeal cardiopulmonary resuscitation
ECMO: Extracorporeal membrane oxygenation
CTM: Cricothyroid membrane
